# Suppression of BRD4 inhibits human hepatocellular carcinoma by repressing MYC and enhancing BIM expression

**DOI:** 10.18632/oncotarget.6275

**Published:** 2015-11-12

**Authors:** Gong-Quan Li, Wen-Zhi Guo, Yi Zhang, Jing-Jing Seng, Hua-Peng Zhang, Xiu-Xian Ma, Gong Zhang, Jie Li, Bing Yan, Hong-Wei Tang, Shan-Shan Li, Li-Dong Wang, Shui-Jun Zhang

**Affiliations:** ^1^ Department of Hepatobiliary and Pancreatic Surgery, The First Affiliated Hospital of Zhengzhou University, Zhengzhou, Henan, China; ^2^ Open and Key Laboratory of Hepatobiliary and Pancreatic Surgery and Digestive Organ Transplantation at Henan Universities, The First Affiliated Hospital of Zhengzhou University, Zhengzhou, Henan, China; ^3^ Department of Orthopaedic Surgery, The First Affiliated Hospital of Zhengzhou University, Zhengzhou, Henan, China; ^4^ The Hormel Institute, University of Minnesota, Minneapolis, MN, USA; ^5^ Department of Pathology, Brigham and Women's Hospital, Harvard Medical School, Boston, MA, USA; ^6^ Department of Pathology, The First Affiliated Hospital of Zhengzhou University, Zhengzhou, Henan, China; ^7^ Henan Key Laboratory for Esophageal Cancer Research, The First Affiliated Hospital of Zhengzhou University, Zhengzhou, Henan, China

**Keywords:** HCC, JQ1, BRD4, BIM, MYC

## Abstract

Bromodomain 4 (BRD4) is an epigenetic regulator that, when inhibited, has anti-cancer effects. In this study, we investigated whether BRD4 could be a target for treatment of human hepatocellular carcinoma (HCC). We show that BRD4 is over-expressed in HCC tissues. Suppression of BRD4, either by siRNA or using JQ1, a pharmaceutical BRD4 inhibitor, reduced cell growth and induced apoptosis in HCC cell lines while also slowing HCC xenograft tumor growth in mice. JQ1 treatment induced G1 cell cycle arrest by repressing *MYC* expression, which led to the up-regulation of *CDKN1B* (*P27*). JQ1 also de-repressed expression of the pro-apoptotic *BCL2L11* (*BIM*). Moreover, siRNA knockdown of *BIM* attenuated JQ1-triggered apoptosis in HCC cells, suggesting an essential role for *BIM* in mediating JQ1 anti-HCC activity.

## INTRODUCTION

Hepatocellular carcinoma (HCC) is one of the most common solid tumors and a leading cause of cancer-related death worldwide [1]. Bromodomain 4 (BRD4) is a member of the bromodomain and extra-terminal (BET) family of proteins [2] which acts as a transcriptional co-activator by facilitating the recruitment of the positive transcription elongation factor P-TEFb [3, 4]. Aberrant BRD4 expression may promote tumorigenesis in multiple myeloma (MM), leukemia and several types of solid cancers via transcriptional activation of oncogenic drivers. Accordingly, there has been much effort to target BRD4 using pharmaceutical inhibitors [3–10].

JQ1 is a small molecule inhibitor of BET proteins with high binding affinity for BRD4 [3]. JQ1 and BRD4 competitively bind to chromatin at gene enhancers, and JQ1 suppresses the expression of oncogenic proteins such as c-Myc [3–10]. Preclinical studies of JQ1 as a treatment for hematological malignancies and solid tumors have demonstrated its anti-cancer activity, largely through the suppression of c-Myc [7–13]. HCC tumors express high levels of c-Myc, and increased c-Myc expression correlates with a more advanced and aggressive phenotype; thus, inhibition of c-Myc has long been proposed as a treatment of HCC [14–20]. In fact, a recent study by Puissant and colleagues reported that several HCC cell lines were sensitive to JQ1 [21].

In this study, we hypothesized that BRD4 inhibitors would act as anti-cancer agents in HCC via c-Myc suppression. To test that idea, we investigated the role of BRD4 in HCC and the anti-cancer effects of BRD4 inhibitors in HCC. We found that BRD4 is over-expressed in HCC tissues and that inhibition of BRD4 by JQ1 suppresses cell proliferation by blocking cell cycle progression and inducing apoptosis in HCC cells.

## RESULTS

### BRD4 is over-expressed in HCC cells and tumor tissues

**Figure 1:**
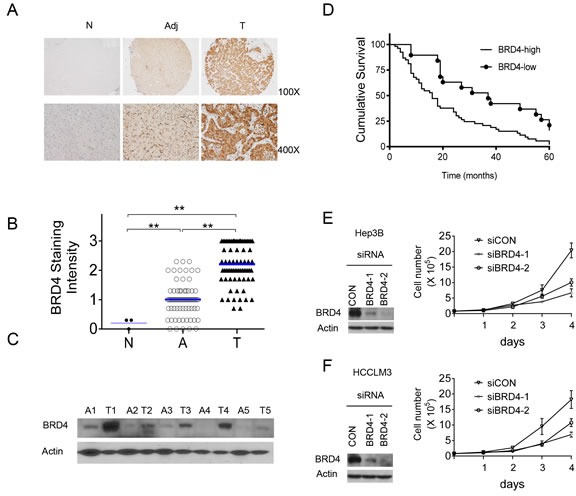
BRD4 is overexpressed in HCC and drives HCC cell growth

We first evaluated BRD4 expression using immunohistochemical (IHC) staining in tissue arrays containing HCC tumors and corresponding adjacent non-neoplastic liver tissues from 72 patients. Normal human liver tissues from three healthy donors were used as controls. BRD4 was weakly expressed in normal liver tissues and highly expressed in both primary tumor and adjacent liver tissues (Figure 1A). However, tumor tissues were stained more intensely than the non-neoplastic liver tissues (*p* < 0.01; Figure 1A, 1B and Figure S1A, S1B). Western blots of 5 pairs of tumors/adjacent liver tissues confirmed increased expression of BRD4 in HCC tumors (Figure 1C). BRD4 expression in HCC tumor tissues differed by and was positively correlated with American Joint Committee on Cancer (AJCC) cancer stage, with mean scores of 1.7 ± 0.2, 2.1 ± 0.1, 2.5 ± 0.1 and 2.6 ± 0.2 for stage I, II, III and IV samples, respectively (one-way ANOVA, *p* < 0.05, Spearman, *p* < 0.05; Figure S1A, Figure S1B, and Table S1). In addition, HCC patients with higher BRD4 expression had lower survival rates than patients with lower BRD4 expression (log-rank test, hazard ratio [HR] = 2.18, 95% confidence interval [CI] = 1.32 to 3.6, *p* < 0.001; Figure 1D). These results suggest that BRD4 expression is increased with HCC disease progression.

**A.**-**D.** BRD4 expression was analyzed by IHC in human liver tissues. **A.** Representative BRD4 staining of de-paraffinized sections of normal liver (N), adjacent liver (Adj) and HCC tumor (T) tissues. **B.** Intensity levels of BRD4 expression are shown for 72 cases of paired adjacent liver and HCC tumor tissues, as well as in normal liver tissues (t-test, A vs. N, **p < 0.01, T vs. N, **p < 0.01, T vs. A, **p < 0.001). **C.** Western blot analysis of BRD4 in HCC tumor tissues (T1-5) and adjacent liver tissues (A1-5). Actin was used as a loading control. **D.** Kaplan-Meier curves for the overall survival rate of patients with HCC according to BRD4 expression (*p <* 0.01, hazard ratio [HR] = 2.18, 95% confidence interval [CI] = 1.32 to 3.6). **E.**, **F.** HCC cell lines Hep3B and HCCLM3 were transfected with two independent BRD4 siRNAs (siBRD4-1, siBRD4-2) or control siRNA (siCON), and the efficiency of BRD4 knockdown was assessed by western blotting analysis 24 h after transfection. Actin was used as a loading control (left panels). The numbers of the transfected cells were measured daily by manual counting for a period of 6 days following transfection (right panels).

### BRD4 expression stimulates HCC cell growth

To investigate the role of BRD4 in HCC cell growth, using two different siRNAs we suppressed *BRD4* expression in Hep3B and HCCLM3 cells and examined cell proliferation. Western blots showed that, in both cell lines, transfection of either siRNA for 24 h led to an almost complete suppression of *BRD4* activation compared with a non-targeting control siRNA (siCON; Figure 1E and 1F). Cells transfected with siCON grew rapidly, whereas cells transfected with BRD4 siRNA had inhibition of cell growth, particularly 4 days post-transfection. These results suggest that BRD4 is essential for HCC cell growth.

### JQ1 reduces human HCC cell proliferation

**Figure 2:**
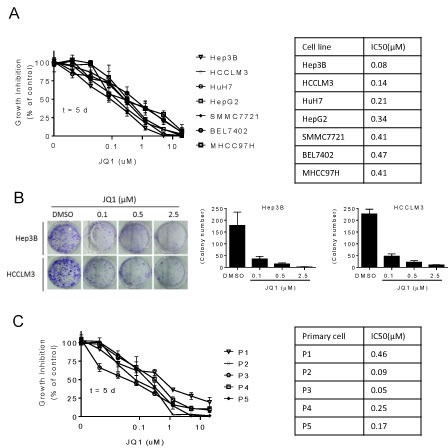
JQ1 inhibits human HCC cell proliferation

Sensitivity to JQ1was evaluated across 7 HCC cell lines by treating cells with serial dilutions of the drug for 5 days and then analyzing cell growth by MTT assays. JQ1 inhibited cell growth in a dose-dependent manner in all 7 HCC cell lines (Figure 2A). Hep3B and HCCLM3 were the most sensitive cell lines with IC50 values of 0.08 and 0.14 μM, respectively (Figure 2A; right panel). Notably, a 5-μM concentration of JQ1 was sufficient to completely inhibit cell growth in these two lines; therefore, they were chosen for subsequent experiments.

We next performed clonogenic assays to determine the long-term anti-proliferative effects of JQ1. JQ1 treatment for 14 days inhibited clone formation in a dose-dependent manner in both cell lines (Figure 2B). Low doses of JQ1 (0.1 μM) reduced clone numbers, while 2.5 μM JQ1 led to an almost complete inhibition of clone formation.

Finally, we examined the inhibitory effect of JQ1 in 5 primary HCC cells freshly isolated from surgically resected tumor tissues. MTT assays revealed that JQ1 treatment for 5 days inhibited primary HCC cell growth in all 5 cases (Figure 2C), with IC50 values observed between 0.05 and 0.5 μM. Together, our results demonstrate that JQ1 reduces proliferation in human HCCs.

**A.** Human HCC cell lines were treated with serial dilutions of JQ1 for 5 days and cell growth was determined using an MTT assay. The dose-response growth curves (left panel) and IC50 values of JQ1 (right panel) were obtained for each cell line. **B.** Hep3B and HCCLM3 cell lines were seeded into six-well plates at 1,000 cells per well in triplicates and treated with JQ1 for 2 weeks, followed by 0.01% (w/v) crystal violet staining and colony counting. Representative images are shown for colony formation for Hep3B and HCCLM3 cell lines (left panels). The data depict the mean ± S.D of clone numbers (right panels). **C.** Primary HCC cells were treated with JQ1 as described in (A), and the dose-response growth curves (left panel) and IC50 values of JQ1 (right panel) were obtained for each cell line.

### JQ1 induces cell cycle arrest and apoptosis in HCC cells

**Figure 3:**
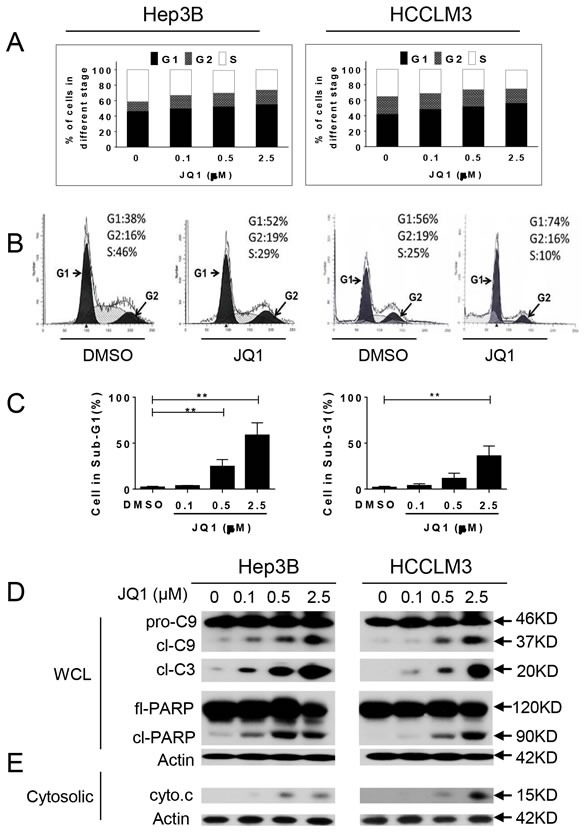
JQ1 arrests cell cycle in the G1 phase and induces apoptosis in HCC cells

To investigate the mechanism underlying the anti-proliferative effects of JQ1 in HCC cells, we analyzed cell cycle distribution using flow cytometry. JQ1 treatment for 48 h led to an increased percentage of HCC cells in G1 phase arrest and a decrease in the percentage of cells in S phase (Figure 3A and 3B). JQ1 treatment also led to a substantial accumulation of HCC cells in sub-G1 phase (Figure 3C) and induced morphological changes characteristic of apoptosis, such as shrinkage, rounding, and floating. These changes became more prominent over time, particularly in Hep3B and HuH7 cells.

Next, we analyzed apoptotic-signaling pathways with cell fractionation and western blotting. JQ1 activated caspase-3 and caspase-9 expression and induced PARP cleavage as well as cytochrome c release into the cytoplasm from mitochondria (Figure 3D and 3E). Caspase-8 expression was not induced by JQ1 (data not shown), indicating that the mitochondrial apoptosis pathway, rather than the extrinsic pathway, mediates the anti-cancer activity of JQ1 in HCC cells. To determine whether caspase-9 was required for JQ1-induced anticancer activity, HCCLM3 and Hep3B cell lines were pre-treated with 50 μM Z-LEHD-FMK, a pharmacological caspase-9 inhibitor for 1 h prior to the addition of JQ1 (2.5 μM) for 3 days (Figure S2). Inhibition of caspase-9 activity reduced JQ1 induction of cell death, suggesting that the caspase-9 initiated mitochondrial apoptosis pathway is essential for JQ1 activity in HCC cells.

**A.**-**C.** Hep3B and HCCLM3 cell lines were treated with JQ1 for 48 h, stained with PI and examined with flow cytometry assays. **A.** The fractions of cells in each phase of the cell cycle are shown for the Hep3B and HCCLM3 cell lines. **B.** Representative plots of the cell cycle phases are shown for Hep3B and HCCLM3 cell lines treated with 0.5 μM JQ1. **C.** The percentage of cells in the subG1 phase is shown for Hep3B and HCCLM3 cell lines. **D.**-**E.** Hep3B and HCCLM3 cells were treated with JQ1 for 48 h. **D.** Hep3B (left panel) and HCCLM3 (right panel) cell lines were treated with JQ1 for 48 h, whole cell lysates were analyzed by western blotting for the expression of pro-caspase-9 (pro-C9), cleaved-caspase-9 (cl-C9), cleaved-caspase-3 (cl-C3), full-length PARP (fl-PARP) and cleaved PARP (cl-PARP). Actin was used as a loading control. **E.** Hep3B (left panel) and HCCLM3 (right panel) cell lines were treated with JQ1 for 48 h, treated cells were fractionated, and the expression of cytochrome c (cyto.c) in the cytosolic compartment was examined by western blotting. Actin was used as a loading control.

### JQ1 represses *MYC* expression and increases p27 expression in HCC cells

**Figure 4:**
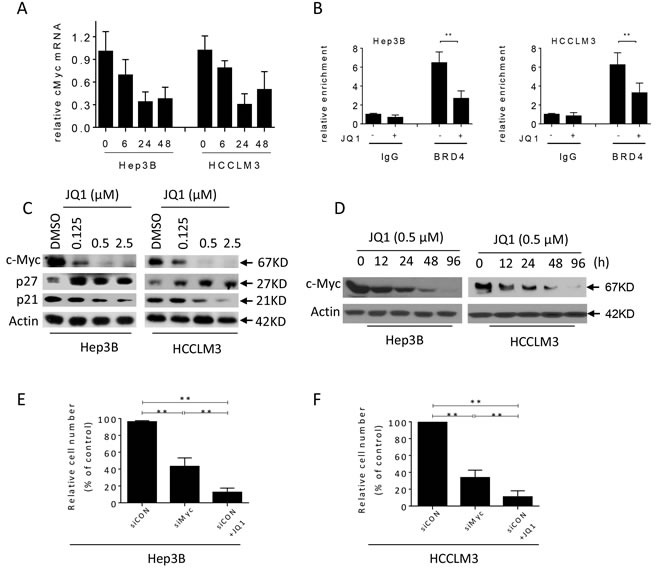
JQ1 suppresses the expression of c-Myc in HCC cells

Building on previous studies which suggested that c-Myc is the critical mediator of JQ1's anti-cancer effects, we next used qRT-PCR and western blots to examine whether JQ1 treatment inhibited c-Myc expression in HCC cells. Indeed, JQ1 inhibited the expression of *MYC* in a time-dependent fashion (Figure 4A). siRNA knockdown of *BRD4* also inhibited *MYC* transcription, suggesting that JQ1 reduced *MYC* through inhibition of BRD4 (Figure S3). To determine whether BRD4 binds directly to the *MYC* gene, we performed a chromatin immunoprecipitation (ChIP) experiment. BRD4 was enriched at the *MYC* enhancer in both HCC cell lines, but this association was reduced after 24 hours of JQ1 treatment (Figure 4B). These results suggest that JQ1 suppresses *MYC* transcription by reducing BRD4 binding to the *MYC* enhancer. Western blots confirmed that transcriptional repression of *MYC* by JQ1 led to time- and concentration-dependent decreases in c-Myc protein levels in both cell lines (Figure 4C and 4D).

Next, using siRNA to *MYC* in HCC cell lines we assessed the effects of this inhibition on cell growth (Figure 4E, 4F and Figure S4). *MYC* knockdown inhibited HCC cell growth; however, the effect was weaker than that triggered by JQ1treatment at 2.5 μM, suggesting that JQ1 may have other functions besides inhibition of c-Myc. Previous studies had reported that JQ1 altered p27 and p21 expression (22, 24); therefore, we investigated the effects of JQ1 on the levels of p27 and p21 in HCC cell lines. We found that JQ1 led to increased p27 expression, but had a strong inhibitory effect on the expression of p21 in both HCC cell lines (Figure 4C and Figure S5).

**A.** Hep3B and HCCLM3 cell lines were treated with 0.5 μM JQ1 for 6, 24 and 48 h. *MYC* mRNA expression was analyzed with qRT-PCR. **B.** Hep3B and HCCLM3 cell lines were treated with 0.5 μM JQ1 for 24 h. Cells were fixed with formaldehyde and subjected to a ChIP assay using antibodies directed against either an IgG control or BRD4. The results are presented as fold enrichment of MYC at the promoter over the control IgG signal. The errors bars represent the standard deviation of three independent experiments (t-test **p < 0.01). The enrichment of GAPDH over IgG signal was used as control in Figure S7. **C.** Hep3B and HCCLM3 cell lines were treated with JQ1 at 0.125, 0.5 and 2.5 μM for 48 h. The expression of c-Myc, p27 and p21 was examined by western blotting analysis in whole cell lysates. Actin was used as a loading control. **D.** Hep3B and HCCLM3 cell lines treated with 0.5 μM JQ1 for 12, 24, 48 and 96 h were harvested for western blot analyses of c-Myc. Actin was used as a loading control. Hep3B **E.** and HCCLM3 **F.** cells were transfected with siControl (siCON) or Myc siRNA in absence or presence of 2.5 μM JQ1 for 96 h. The relative cell number was manually determined in each condition (t-test, **p < 0.01).

### BIM is required for JQ1-induced anti-cancer effects in HCC cells

**Figure 5:**
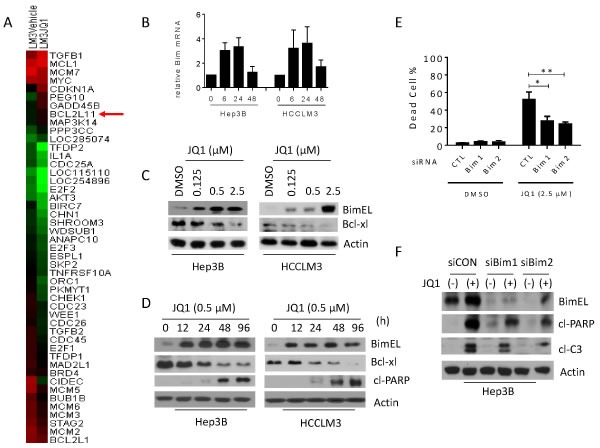
JQ1-induced anti-cancer activity requires Bim up-regulation in HCC cells

To unravel the discrepancy in the severity of anti-cancer effects observed between HCC cells treated with c-Myc siRNA or JQ1 (Figure 4E and 4F), we performed RNA-Seq analysis in HCCLM3 cells treated with or without 0.5 μM JQ1 for 4 h. Acute JQ1 treatment led to changes in the expression of a large number of genes, and the complete results are provided online (Supporting Information). A heat map depicting the top 46 genes that were altered post-JQ1 treatment revealed enrichment for genes involved in the cell cycle and/or apoptosis (Figure 5A). As expected, *MYC* was among the genes inhibited by JQ1 treatment. In addition, the pro-apoptotic gene *BCL2L11* (*BIM*) was increased more than 2-fold after acute JQ1 treatment (Figure 5A and Supporting Information), a finding that was confirmed by qRT-PCR. Peak levels of *BIM* mRNA were observed after 6 h of JQ1 treatment and were sustained for at least 48 h in both cell lines (Figure 5B). Western blots validated that de-repression of *BIM* results in a dramatic increase in BIM protein levels in both HCC cell lines (Figure 5C and 5D). siRNA knockdown of BRD4 also increased *BIM* transcription, suggesting that JQ1 enhances *BIM* expression through inhibition of BRD4 (Figure S3).

Because BIM is a potent pro-apoptotic BCL-2 protein that binds to and antagonizes several key anti-apoptotic family members [25, 26], we hypothesized that BIM is involved in JQ1-induced apoptosis. siRNA knockdown of *BIM* reduced JQ1-induced cell death by 50% in HCC cell lines (Figure 5E and Figure S6). Moreover*, BIM* knockdown attenuated JQ1-induced caspase-3 activation and PARP cleavage (Figure 5F), suggesting that BIM is required for JQ1-triggered apoptosis in HCC cells.

**A.** HCCLM3 cells were treated with 0.5 μM JQ1 or DMSO control for 4 h. JQ1-induced changes in global gene expression were examined by RNA-Seq analysis. A heat map depicting the top 46 inhibited or increased (p ≤ 0.01 and fold change ≥ 1.5 log2) genes display enrichment for genes involved in regulation of cell cycle and apoptosis. **B.** Hep3B and HCCLM3 cell lines were treated with 0.5 μM JQ1 for 6, 24 and 48 h. *BIMEL* mRNA expression was analyzed using qRT-PCR. **C.** Hep3B and HCCLM3 cell lines were treated with JQ1 at 0.125, 0.5 and 2.5 μM for 48 h. The expression of BIMEL was examined by western blotting analysis of whole cell lysates. Actin was used as a loading control. **D.** Hep3B and HCCLM3 cell lines were treated with 0.5 μM JQ1 for 12, 24, 48 and 96 h, and the cells were harvested for western blot analysis of BimEL and cleaved PARP (cl-PARP). Actin was used as a loading control. **E.**, **F.** Hep3B cell line was transfected with control siRNA or Bim siRNA (Bim1 and Bim2) for 24 h. Transfected cells were treated with 2.5 μM JQ1 for another 72 h, **E.** cell death was examined with trypan blue exclusion assays (t-test, *p < 0.05). **F.** The expression of BimEL, cleaved PARP (cl-PARP) and cleaved Caspase-3 (cl-C3) was examined with western blotting analysis. Actin was used as a loading control.

### JQ1 inhibits tumor growth in HCC mouse models

**Figure 6:**
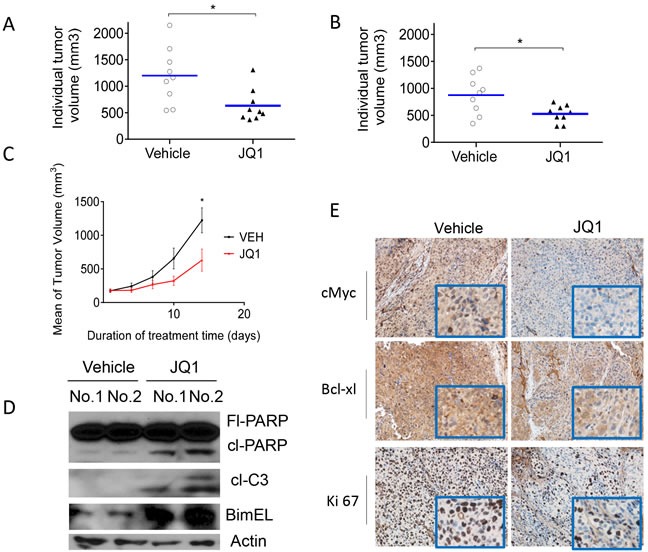
JQ1 inhibits tumor growth in subcutaneous HCC models

To investigate the anti-tumor effects of JQ1 *in vivo*, we employed HCCLM3 and Hep3B HCC xenograft models. HCC cells were injected subcutaneously into the flanks of nude mice, and, after palpable xenograft tumors were established, the mice were randomly assigned into treatment or control groups. For the treatment groups, JQ1 was administered intra-peritoneally to mice twice daily at 50 mg/kg body weight for a total of 14 days. The average tumor volumes in the JQ1-treated groups were smaller than those in the vehicle groups on day 14 in both models (Hep3B, *p* < 0.05, HCCLM3, *p* < 0.05; Figure 6A and 6B). Moreover, analysis by two-way ANOVA revealed that JQ1 treatment delayed HCC xenograft growth in the Hep3B model (*p* < 0.05; Figure 6C). Mice treated with JQ1 showed no obvious signs of toxicity (based on body weight, food and water intake, activity and general examination) during treatment.

JQ1 treated and untreated tumor tissues from the Hep3B xenograft model were analyzed by western blots and IHC. Treatment with JQ1 at 50 mg/kg twice per day for 3 days led to BIM accumulation, PARP cleavage, and caspase-3 activation, suggesting the activation of apoptotic signaling in tumor tissues (Figure 6D). JQ1 treatment also inhibited c-Myc and Ki-67 expression in HCC tumor tissues (Figure 6E). Together, these data suggest that JQ1 promotes anti-tumor activity *in vivo* by inhibiting cell cycle regulators and activating pro-apoptotic signaling pathways.

**A.**-**C.** Hep3B and HCCLM3 cell lines were implanted subcutaneously in mice and allowed to grow until the tumors reached a size of approximately 150 mm3. Xenografted mice were randomized and treated with JQ1 at 50 mg/kg i.p. or vehicle twice daily (BID) 5 times a week for 2 weeks. Tumor volume was monitored during the treatments. **A.** The graph shows changes in the volume of Hep3B tumors. The data are the mean ± SE (n = 9). Tumor volumes from two groups were analyzed by two-way ANOVA analysis (**p < 0.05). **B.** The graph shows individual tumor volume of Hep3B tumors (t-test, *p < 0.05). **C.** The graph shows individual tumor volume of HCCLM3 tumors (t-test, *p < 0.05). **D.**-**E.** Hep3B tumors were excised and harvested from animals treated with JQ1 at 50 mg/kg i.p. BID or vehicle for 3 days. **D.** The levels of BIMEL, fl-PARP, cl-PARP, and cl-C3 in tissue lysates were examined with western blotting analysis. Actin was used as a loading control. **E.** The expression of c-Myc and Ki 67 in HCC xenograft tissues was examined by IHC analyses. Representative images are shown for vehicle and treated tumors.

## DISCUSSION

In the present study, we found that BRD4 expression was increased in HCC cell lines and tumor tissue and correlated with HCC disease progression. Moreover, we showed that suppression of BRD4 using siRNAs inhibited HCC cell growth. These data suggest a role for BRD4 in HCC development and survival and are in agreement with recent results by Zhang et al, which showed that forced expression of BRD4 promotes cell growth, while shRNA-induced suppression of BRD4 inhibits HCC cell growth [23]. We further showed that JQ1 arrested cell cycle progression at G1 phase, induced apoptosis, and inhibited clonogenic survival and growth at low concentration ranges in HCC cell lines and patient-derived primary HCC cells. Finally, using mouse HCC xenograft tumor models, we showed that 2 weeks of treatment with JQ1 inhibited tumor growth by suppressing proliferation and inducing apoptosis without severe toxicity. These data suggest that inhibition of BRD4 with small molecule inhibitors such as JQ1 represents a novel strategy for the treatment of HCC.

We also elucidated the mechanism by which JQ1 blocks cell cycle progression in HCC cells and observed that JQ1 treatment increased p27 expression in HCC cells. Because p27 represents a key downstream mediator of c-Myc-induced regulation of cell proliferation [24, 27], our findings suggest that the JQ1-induced cell cycle arrest may rely on a BRD4-c-Myc-p27 axis in HCC cells. Surprisingly, we found that JQ1 treatment reduced p21 levels in HCC cells, which is in contrast to previous reports that JQ1 arrests the cell cycle at the G1 phase by upregulation of p21 in non-small cell lung cancer, glioblastoma, MM cells, and acute myeloid leukemia cells [10, 11, 24–30]. It is also important to note that the inhibitory effects of JQ1 on c-Myc transcription appeared somewhat delayed in HCC cells compared with kinetics previously observed in MM cells [22]. This suggests regulation of gene transcription by BRD4 is cell type-dependent

Our analysis of the mechanisms underlying apoptosis revealed that the mitochondrial apoptosis pathway is involved in JQ1-induced anti-HCC effects. This conclusion is supported by western blot analysis, which showed that JQ1 induces PARP cleavage, caspase-3 activation, and cytosolic cytochrome c release in HCC cells. Mitochondrial apoptosis is promoted by the pro-apoptotic BCL-2 family of proteins [31]. In this study, we unexpectedly observed that JQ1 treatment led to rapid and dramatic BIM up-regulation at both the mRNA and protein level in HCC cells. Moreover, our data show that BIM suppression rescues HCC cells from JQ1-induced apoptotic cell death, suggesting an essential role for BIM in the anti-HCC effects of JQ1.

Recently, other groups have reported that BET inhibition leads to upregulation of BIM. Using a malignant peripheral nerve sheath tumors (MPNSTs) mouse model, Patel and colleagues showed that suppression of BRD4 with either JQ1 or siRNA rapidly increases BIM expression in MPNSTs cells and, similar to the results presented here, *BIM* knockdown inhibits JQ1-induced apoptosis [26]. In addition, Gallagher and colleagues showed that BIM is required for the induction of apoptosis by the BET protein inhibitor I-BET151 in melanoma cells [25]. Together, these studies underscore the importance of BIM in the induction of apoptosis resulting from BRD4 (BET) inhibition. As BRD4 regulates gene expression through the recruitment of the P-TEFb, it remains to be determined how JQ1 inhibition of BRD4 de-represses BIM expression. Moreover, because JQ1 also inhibits BRD2, BRD3, and other BET family members, in the future it will be important to test the extent to which BET family members contribute to JQ1 anti-tumor activity in HCC and other cancers [4, 32].

Our finding that BRD4 expression is elevated in a fraction of adjacent non-tumor liver tissue, albeit to a lesser extent than in tumor tissue, raises a potential concern for the future clinical utility of BRD4 inhibitors in human HCC, as improper drug use may cause liver damage. In addition, although JQ1 stimulated anti-HCC activity *in vitro*, treatment with JQ1 at 50 mg/kg twice daily *in vivo* had only a modest effect on tumor growth, suggesting that BRD4 inhibitors with increased bioavailability or potency may be required for improved therapeutic responses [33].

## MATERIALS AND METHODS

### Cell lines and compound preparation

The HCC cell lines Hep3B, HCCLM3, HuH7, HepG2, MHCC97H, SMMC7721 and BEL7402 were obtained from the China Center for Type Culture Collection (Wuhan, China) and maintained in high-glucose DMEM (HyClone/Thermo Fisher Scientific, Beijing, China) supplemented with 10% heat-inactivated fetal bovine serum (Hangzhou Sijiqing Biological Engineering Materials Co., Ltd, Hangzhou, China). JQ1 was kindly gifted by Professor James Bradner (Harvard Medical School). JQ1 was dissolved in Dimethyl sulfoxide (DMSO) at a stock concentration of 10 mmol/L and stored at −20°C.

### MTT cell viability assay

Cell viability was measured using a 3-[4,5-dimethylthiazol-2-thiazolyl]-2,5-diphenyl-tetrazolium bromide (MTT) assay that is based on mitochondrial conversion of MTT from a soluble tetrazolium salt into an insoluble colored formazan precipitate, which was dissolved in DMSO and quantified by spectrophotometry (Thermo Multiskan MK3; Thermo Labsystems, Shanghai, China) to obtain optical density (OD) values. HCC cells were plated in 96-well culture dishes (Costar, Cambridge, MA, USA) at a density of 1000-2000 cells/well in 100 μL of medium. Serial dilutions were generated from a stock solution of JQ1 to the desired concentrations. All experimental concentrations were replicated in triplicate. Four hours before the desired time points, 10 μL of 10 mg/mL MTT was added. After a 4-h incubation, all media from wells were removed, and 100 μL of DMSO was added. The percentages of absorbance relative to those of untreated control samples were plotted as a function of drug concentration (log scale). Inhibition of cell viability was measured by percentage of viable cells relative to the control: % inhibition = 100% × ODT / ODC, where ODT is the average OD value of the treated samples and ODC is the average OD value of the control samples.

### Cell death, flow cytometry, and clonogenic assays

Cell death was quantified by microscopic examination in trypan blue exclusion assays. Cell cycle analysis was examined by propidium iodide (PI, 50 μg/mL in PBS) and flow cytometry with a BD LSR II system (BD Biosciences, Shanghai, China). For clonogenic assays, 1,000 cells were seeded into 6-well dishes in 5 mL of medium, treated as indicated, and maintained for 14 days at 37°C in a 5% CO2 incubator. Cells were then washed with drug-free medium, stained with 0.01% (w/v) crystal violet, and cell colonies (> 50 cells) were counted at 14 days post-treatment. The assays were performed in duplicate with at least three different repeats per treatment.

### Cell fractionation

HCC cells were treated as indicated, collected, washed with PBS and suspended in 5 volumes of chilled buffer A (250 mM sucrose, 20 mM HEPES, 10 mM KCl, 1.5 mM MgCl2, 1 mM EDTA, 1 mM EGTA, 1 mM DL-dithiothreitol [DTT], 17 μg/mL phenylmethylsulfonyl fluoride [PMSF], 8 μg/mL aprotinin and 2 μg/mL leupeptin [pH 7.4]) on ice for 15 min. Cell fractionation was performed using the homogenization method. Briefly, cells were homogenized using an ice-cold cylinder cell homogenizer (20-25 strokes). Homogenized cell lysates were separated by centrifugation at 750 g for 10 min, and the supernatants were further centrifuged at 10,000 g for 20 min. The remaining supernatant was used as the cytosolic fraction and subjected to western blot analysis.

### Western blotting

Cells were lysed using radioimmunoprecipitation (RIPA) assay lysis buffer (PBS containing 1% NP40, 0.5% Na-deoxycholate, and 0.1% SDS) supplemented with 1 μmol/L phenylmethylsulfonyl fluoride and 1 protease inhibitor cocktail tablet per 10 mL on ice for 20 min, and lysates protein concentration were determined using the Bio-Rad protein assay kit according to the manufacturer's instructions. Proteins were electrophoresed onto 4-20% SDS-PAGE gels (Invitrogen, Carlsbad, CA, USA) and transferred onto polyvinylidene difluoride membranes. Following blocking in 5% milk, the membranes were incubated with a specific primary antibody, washed, and incubated with horseradish peroxidase-linked secondary antibody (GE Healthcare, Beijing, China). Signals were visualized with chemiluminescent horseradish peroxidase antibody detection reagent (Denville Scientific, Guangzhou, China).

The antibodies used were as follows: BRD4 (H-250)(sc-48772); Caspase-9 (96.1.23)(sc-56076); Caspase-3 (H-277)(sc-7148); c-Myc (9E10)(sc-40); p27 (C-19)(sc-528); p21 (C-19)(sc-397); Mcl-1 (S-19)(sc-819); Bim (H-191) (sc-11425); and Actin (C4)(sc-47778). The antibodies were purchased from Santa Cruz Biotechnology (Shanghai, China).

### qRT-PCR

Total RNA was isolated using TRIzol reagent (Invitrogen), and cDNA was synthesized using the high capacity cDNA archive kit (Applied Biosystems). The mRNA level of *MYC* was quantified by qRT-PCR using SYBR Premix Ex Taq (Applied Takara Bio, Shanghai, China). The *MYC* primers were as follows: 5′-TCTCCACTCACCAGCACAACTACG and 3′-ATCTGCTTCAGGACCCT. The *BIM* (*BCL2L11*) primers were as follows: 5′- TAAGTTCTGAGTGTGACCGAGA-3′ and 3′- GCTCTGTCTGTAGGGAGGTAGG-5′. The *CDKN1A (p21)* primers were as follows: 5′- GAGGCCGGGATGAGTTGGGAGGAG-3′, 3′-CAGCCGGCGTTTGGAGTGGTAGAA-5. The *CDKN1B (p27)* primers were as follows: 5-CCGGTGGACCACGAAGAGT-3, and 5-GCTCGCCTCTTCCATGTCTC-3. The *GAPDH* primers were as follows: 5-TGCCTCCTGCACCACCAACT-3, and 5- CGCCTGCTTCACCACCTTC-3. The PCR conditions included an initial denaturation step of 95°C for 2 min, followed by 35 cycles of 95°C for 10 s, 56°C for 20 s, and 72°C for 20 s, and a final elongation step of 72°C for 10 min. Quantitation relative to the endogenous control (*GAPDH*) was performed using the Applied Biosystems 7500 Fast System SDS software.

### RNA interference

Commercially available siRNAs inhibiting BRD4 and BIM were used to knock down the respective proteins in HCC cell lines. The siRNA transfections (50 pmol/L) were performed using Lipofectamine RNAiMax transfection reagent (Invitrogen, Shanghai, China). Non-silencing siRNAs (siCON) and validated BRD4 siRNAs (siBRD4-1) were purchased from Qiagen. BRD4 siRNA (siBRD4-2) (sc-43639) and BIM siRNA (siBim-1) (sc-29802) were purchased from (Santa Cruz Biotechnology), the second BIM siRNA (siBim2) (6461) was purchased from Cell Signaling Technology (Shanghai, China), and c-Myc siRNA (siMYC) were purchased from (GE Dharmacon, Shanghai, China).

### Chromatin-immunoprecipitation (ChIP)

ChIP experiments were conducted as described previously (13). Briefly, prior to harvesting, sodium butyrate (Sigma-Aldrich) was added to the cell culture medium to a final concentration of 20 mM and mixed gently. The cells were then harvested by trypsinization and cross-linked with 1% (v/v) formaldehyde for 10 min at room temperature. The cross-linking reaction was terminated by adding glycine to a final concentration of 125 mM for 5 min. Cells were then lysed with lysis buffer (50 mM Tris-HCl, 10 mM EDTA, 1% SDS, protease inhibitor cocktail and 20 mM sodium butyrate). The chromatin was sheared by sonication to a DNA fragment size of 200-600 bp and precipitated by centrifugation at 12 000 × g at 4°C for 10 min. For immunoprecipitations, 5 μg of BRD4 antibody (Cell Signaling Technology, #13440) or of a rabbit immunoglobulin G (IgG) (Cell Signaling Technology, #2729) control were incubated overnight with the sheared DNA. The next day, 50 μL of protein G Dynabeads (Life Technologies) were added and incubated for 4 h at 4°C. The antibody/chromatin/beads complexes were washed four times with RIPA buffer (10 mM Tris-HCl, 1 mM EDTA, 0.5 mM EGTA, 1% Triton-X100, 0,1% SDS, 0,1% sodium-deoxycholate and 140 mM NaCl) and once with TE buffer (10 mM Tris-HCl, 10 mM EDTA). DNA was incubated with elution buffer (20 mM Tris-HCl, 5 mM EDTA, 20 mM sodium butyrate, 50 mM NaCl) containing 50 μg/mL proteinase K at 68°C for 2 h. DNA was purified using the Qiagen MinElute columns (Qiagen). For all reactions, 300 pg of ChIP and input DNA were analyzed using qPCR with the following primers (4, 5): *MYC* promoters (forward) 5′-TCACGTTTGCCATTACCGGTTC-′3 and (reverse) 5′-TTTCAGGTTGGCTGCA G A AGGT-3′. Promoters for *GAPDH*: 5-TGCCTCCTGCACCACCAACT-3, and 5- CGCCTGCTTCACCACCTTC-3. were used to rule out non-specific binding (Figure S7).

### Primary HCC cell isolation, culture and treatment

Fresh HCC tissues were obtained from the surgical specimens at the Department of Hepatobiliary and Pancreatic Surgery of the First Affiliated Hospital of Zhengzhou University as described previously [34]. Written informed consent was obtained from each patient, and the study was approved by the Ethics Committee of the First Affiliated Hospital of Zhengzhou University based on the ethical guidelines of the 1975 Declaration of Helsinki.

### Immunohistochemistry (IHC)

A tissue microarray was obtained from Shanghai Outdo Biotech Co., Ltd (Shanghai, China). Normal liver tissue sections were obtained from the Pathology Department of the First Affiliated Hospital of Zhengzhou University with patient consent. Tumor tissues were obtained from tumor-bearing mice treated with JQ1 or vehicle control for 3 days. The antibodies used for IHC were from the following companies: BRD4 (HPA015055) from Sigma (Shanghai, China); c-MYC (AF3696) from R&D systems (Shanghai, China); and Ki 67 (550609) from BD Biosciences (Shanghai, China). IHC was performed as described previously [6]. Briefly, the sections were de-paraffinized by xylene, rehydrated in graded concentrations of ethanol and boiled in antigen retrieval buffer (Abcam, Shanghai, China) in a microwave oven for 5 min. Slides of consecutive sections were incubated with diluted antibodies at room temperature for 2 h. After incubation, the slides were washed three times with PBS, incubated with horseradish peroxidase (HRP)-conjugated antibody (Invitrogen, Shanghai, USA) at room temperature for 30 min, followed by incubation of ABC (avidin-biotin complex, Vectorlabs, Shanghai, China) for 30 min and visualization by the addition of 3,3′-diaminobenzidine tetrahydrochloride (DAB) reagent (Dako Diagnostics (Shanghai) Co., Ltd.), with hematoxylin as the counter stain. Images of stained slides were captured using a standard light microscope.

### Animal studies

All experimental procedures were performed in accordance with protocols approved by the Institutional Laboratory Animal Care and Use Committee of Zhengzhou University. All animals received humane care according to the criteria outlined in the “Guide for the Care and Use of Laboratory Animals Chinese Version” (2006). HCC Hep3B and HCCLM3 cells (5 × 106 cells) suspended in 0.1 mL of Matrigel were implanted subcutaneously into the flank of 5-week-old athymic nude mice (Hunan Slack King of laboratory animals, Changsha, China). Once tumors had reached a minimal volume of 100 mm3, the mice were randomly assigned to the control or treated groups (nine to 10 mice per group). Tumor-bearing mice were treated twice (BID) per day with JQ1, 50 mg/kg, i.p. or an equal amount vehicle as a control for 2 weeks. Tumor volumes were evaluated twice weekly by measuring two perpendicular diameters with calipers. Tumor volume (V) was calculated using the following equation: V = (a2 x b), where a is the width of the tumor (small diameter) and b the length (large diameter) in millimeters. To investigate the mechanism of JQ1-induced anti-tumor activity, tumor-bearing mice were treated twice per day with JQ1, 50 mg/kg, i.p, or vehicle for 3 days. Then, the mice were sacrificed, and tumor tissues were harvested and either fixed in formalin for IHC staining for c-MYC and Ki 67 or lysed and examined for PARP cleavage and caspase-3 activation.

### RNA-Seq

Cells were treated with JQ1 or the corresponding DMSO control for 4 h. RNA-Seq expression analysis was performed by BGI-Tech Company (Shenzhen, China). Complete results are reported in Supplementary File 1 (http://www.ncbi.nlm.nih.gov/bioproject/281518,PRJNA281518).

### Statistical analyses

All data are displayed as the mean ± SEM unless specified otherwise. The types of statistical methods that were used to evaluate statistical significance (*p* < 0.05 was deemed significant) are indicated in the text and/or figure legends.

## SUPPLEMENTARY MATERIAL TABLE AND FIGURES


