# Supplementary material for: Suppression of BRD4 inhibits human hepatocellular carcinoma by repressing MYC and enhancing BIM expression
Source: Oncotarget. 2015 Nov 12;7(3):2462–74. doi: 10.18632/oncotarget.6275 (PMC4823048; doi:10.18632/oncotarget.6275)
Supplement: Supplementary file 1 [file oncotarget-07-2462-s001.pdf]

Suppression of BRD4 inhibits human hepatocellular carcinoma by repressing MYC and enhancing BIM expression

Supplementary Material

Supplementary (S) Table 1: Association of BRD4 expression with patient's clinicopathologic characteristics in HCC

Variable		BRD4 expression		
		High score	Low score	p
Age				
≥60	18	3	15	$p > 0.05$
<60	54	13	41	
Gender				
Male	60	18	42	$p > 0.05$
Female	12	3	9	
AJCC stage				
I	16	7	9	$p < 0.05$
II	26	20	6	
III	22	19	3	
IV	8	7	1	
AFP (ng/ml)				
≥100	46	32	14	$p > 0.05$
<100	26	21	5	
Blood vessel invasion				
negative	51	31	20	$p < 0.01$
positive	21	19	2	
Lymph node metastasis				
negative	64	46	18	$p > 0.05$
positive	8	7	1	

Fig. S1A

Expression of BRD4 in adjacent tissues (A) and tumor tissues (T).

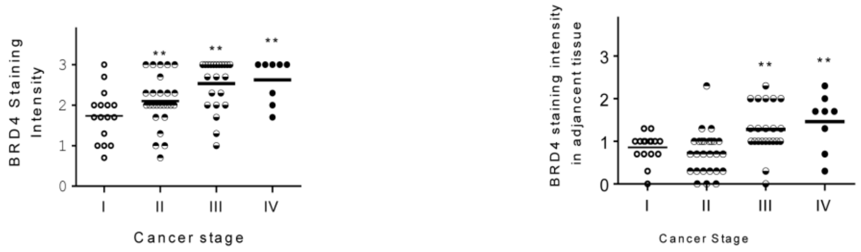

Figure legend: IHC staining of BRD4 expression was performed on tissue arrays, and the staining intensity was graded on a scale of 0-3. (left panel) The score of BRD4 intensity of individual tumor was plotted according to cancer stage. One-way ANOVA analysis was used to determine the statistically difference between different groups, and Spearman analysis was used to determine the correlation between staining intensity and cancer stage. (One-way ANOVA, **$p < 0.01$; Spearman correlation analysis, **$p < 0.01$). (right panel) The score of BRD4 intensity of individual adjacent liver tissue was plotted according to AJCC (American Joint Committee on Cancer) cancer stage. (One-way ANOVA, **$p < 0.01$; Spearman correlation analysis, **$p < 0.01$).

Fig. S1B

Complete photographs of BRD4 expression in HCC tumor tissues (T) and corresponding adjacent tissues from different stage of HCC in next 6 slides.

Figure legend for next 6 slides: IHC was performed on tissue arrays, and a photograph was shown for individual HCC tumor tissue and its corresponding adjacent liver tissues according to cancer stages (from SI Fig.2 (T1) to SI Fig.2 (T4)). Based on staining intensity, a score was given to each sample by three trained pathologists from the weakest (0) to the strongest (3), and the average score number was shown in individual photograph.

Fig. S1B (T-1) Photographs of BRD4 expression in tumor tissues (T) and adjacent tissues (A) obtained from stage T1.

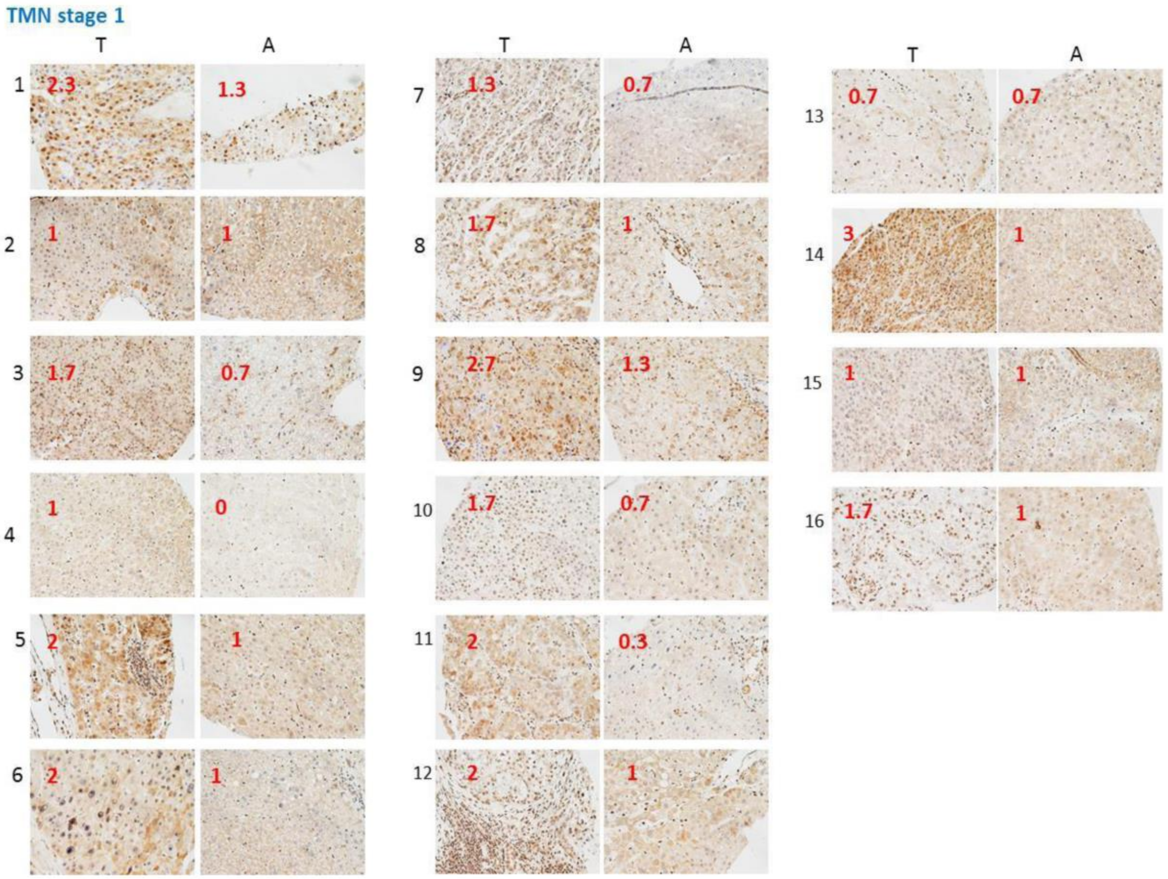

Fig. S1B (T-2a) Photographs of BRD4 expression in tumor tissues (T) and adjacent tissues (A) obtained from stage T2.

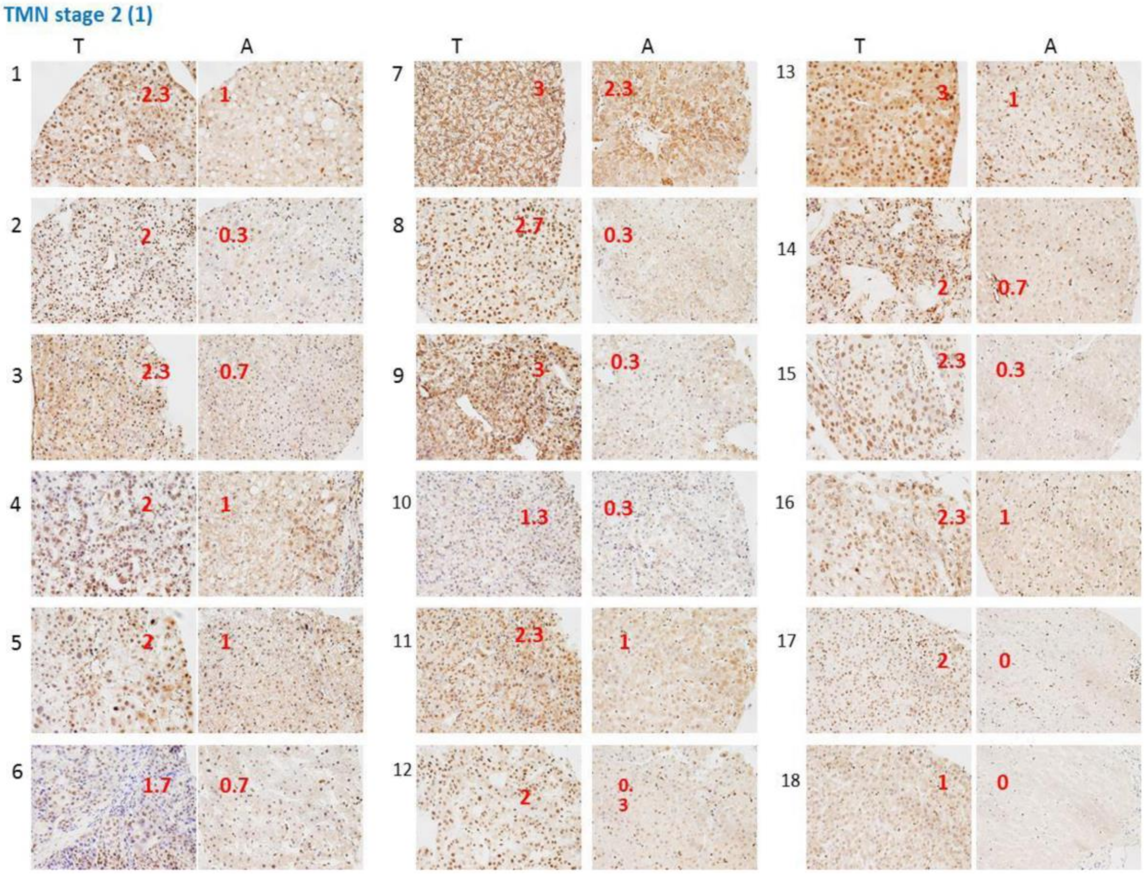

Fig. S1B (T-2b) Photographs of BRD4 expression in tumor tissues (T) and adjacent tissues (A) obtained from stage T2.

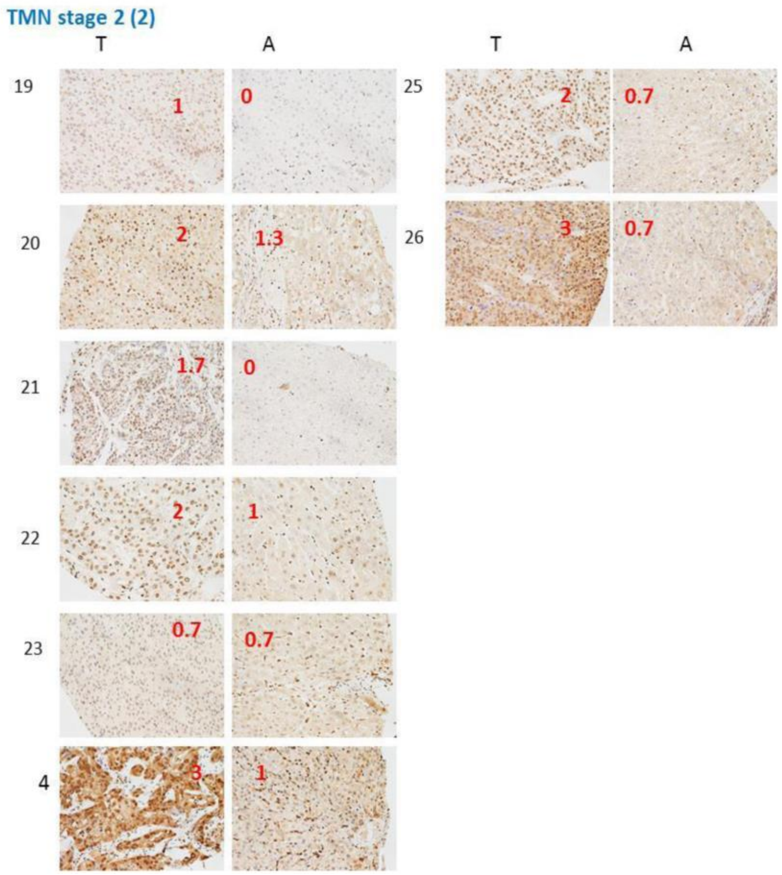

Fig. S1B (T-3a) Photographs of BRD4 expression in tumor tissues (T) and adjacent tissues (A) obtained from stage T3.

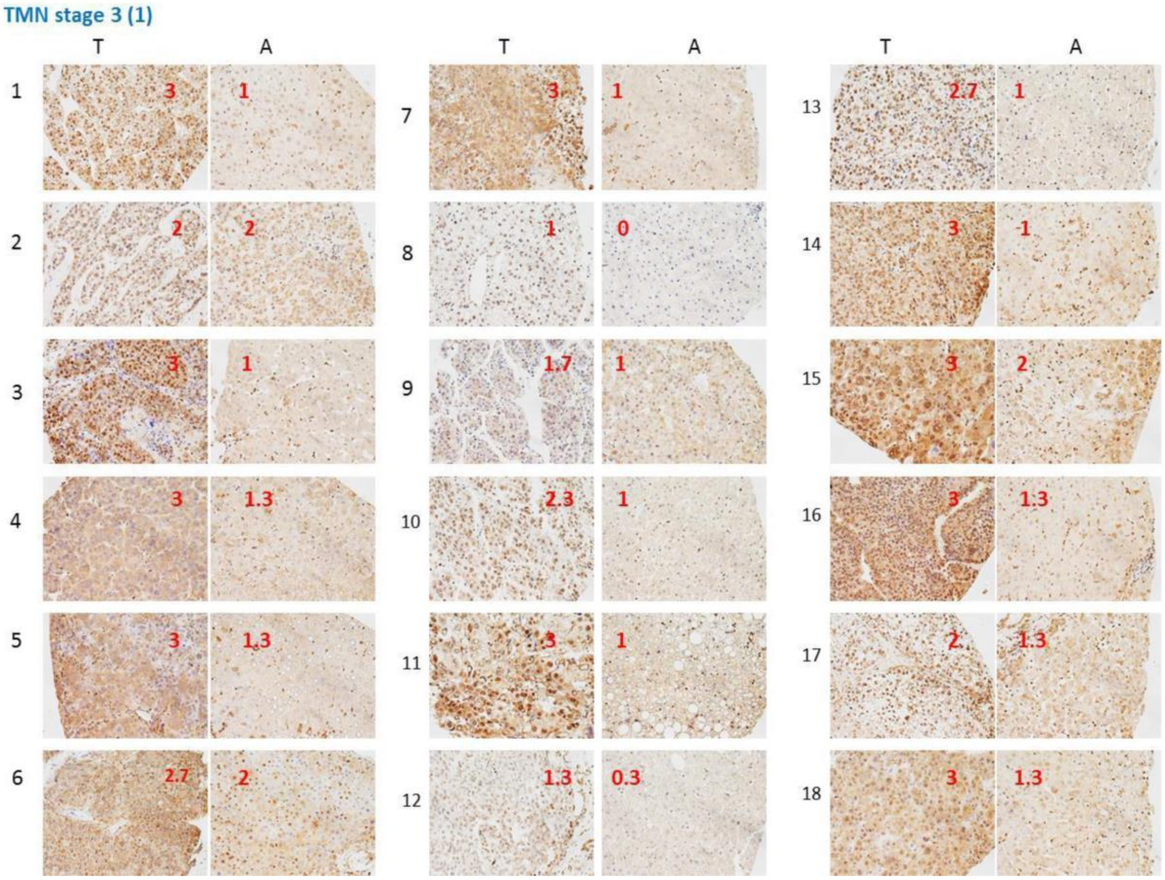

Fig. S1B (T-3b) Photographs of BRD4 expression in tumor tissues (T) and adjacent tissues (A) obtained from stage T3.

TMN stage 3 (2)

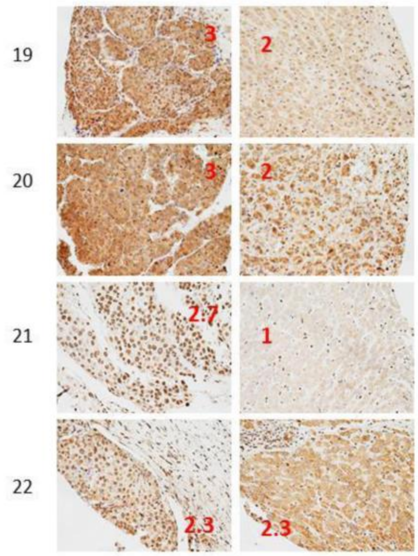

Fig. S1B (T-4) Photographs of BRD4 expression in tumor tissues (T) and adjacent tissues (A) obtained from stage T4.

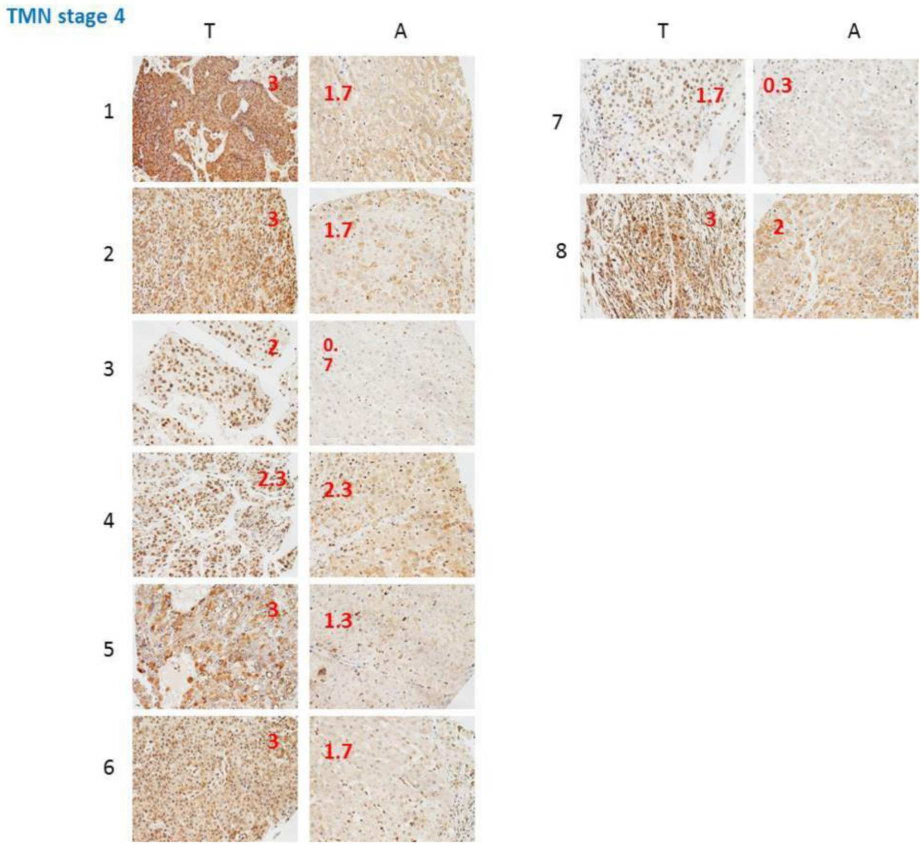

Fig. S2 JQ1-mediated anticancer activity is dependent on caspase-9 in HCC cells.

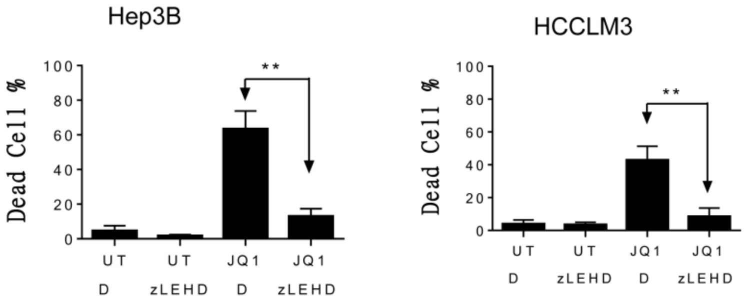

Figure legend: (A) Hep3B and (B) HCCLM3 cell lines pretreated with caspase-9 inhibitor (Z-LEHD.fmk) at 50 μM for 1 h before treatment with JQ1 at 2.5 μM for 72 h, cell death induction was examined with a trypan blue exclusion assay. (t-test, **$p < 0.01$).

Fig. S3 Knockdown of BRD4 increases Bim expression and suppresses MYC expression in Hep3B cell line.

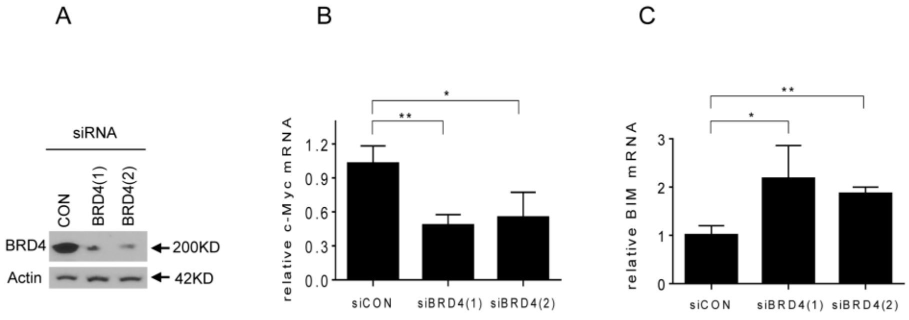

Figure legend: (A) Hep3B cell line was transfected with siRNA against BRD4 for 2 days, and the transfection efficiency was determined by western blotting analysis. Hep3B cell line was transfected with siRNA against BRD4 for 4 days, The relative mRNA expression levels of c-Myc (left panel) and BIM (right panel) were analyzed with qRT-PCR.

Fig. S4 Inhibition of cMyc contributes in part to JQ1-mediated anticancer activity in HCC Hep3B cell line.

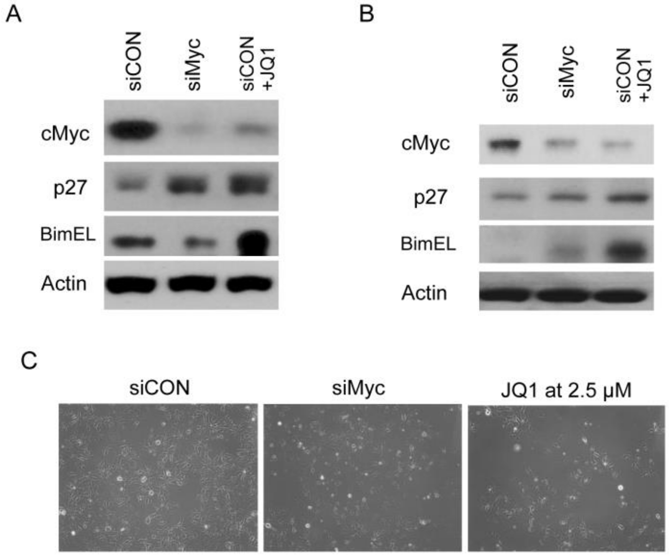

Figure legend: Hep3B cell line (A) and HCCLM3 (B) were transfected with siControl (siCON) or Myc siRNA (siMyc) in absence or presence of JQ1 at 2.5 μM for 48 h. (A) Transfected effectiveness and the effect on p27 and BimEL level were examined by western blotting analysis. Actin was used as a loading control. (C) Hep3B cell line was transfected with siCON (C) or Myc siRNA in absence or presence of JQ1 at 2.5 μM for 96 h, representive photographs were shown for each conditions.

Fig. S5 JQ1 enhances the transcription of p27, while inhibits the transcription of p21 in HCC cells.

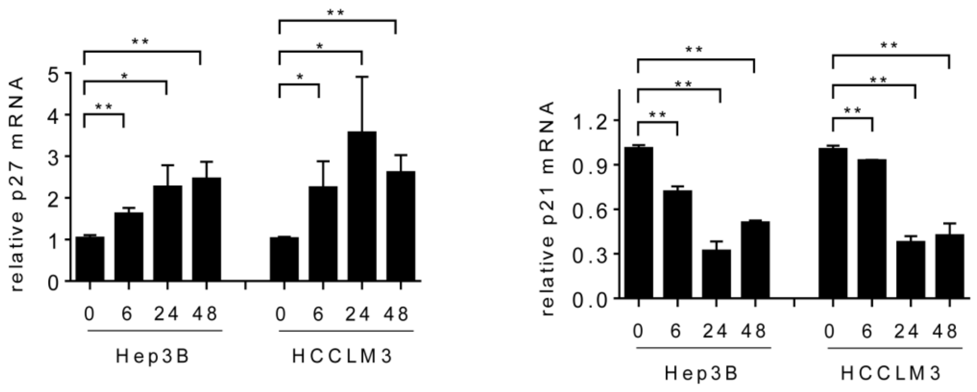

Figure legend: Hep3B and HCCLM3 cell lines were treated with 0.5 µM JQ1 for 6, 24 and 48 h. The relative mRNA expression levels of p27 (left panel) and p21 (right panel) were analyzed with qRT-PCR.

Fig. S6 Bim contributes essentially to JQ1-mediated anticancer activity in HCCLM3 cell line.

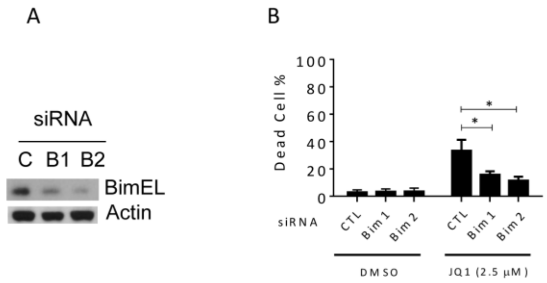

Figure legend: HCCLM3 cell line was transfected with control siRNA or Bim siRNA (B1 and B2) for 24 h. Transfected effectiveness was examined by western blotting analysis. Actin was used as a loading control. (E) Transfected cells were treated with JQ1 at 2.5 μM for another 72 h, cell death was examined with trypan blue exclusion assays. (test, $*p < 0.05$).

Fig. S7

There is no enrichment of GAPDH over the control IgG signal in CHIP analysis.

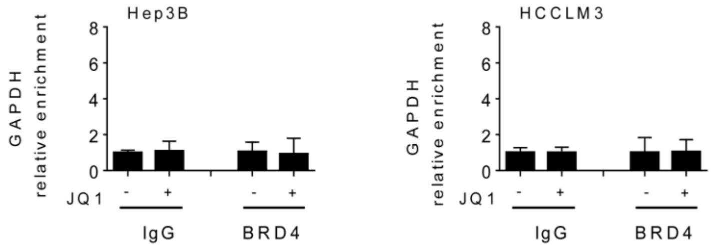

Figure legend: Hep3B and HCCLM3 cell lines were treated with 0.5 μM JQ1 for 24 h. Cells were fixed with formaldehyde and subjected to a ChIP assay using antibodies directed against either an IgG control or BRD4. The results are presented as fold enrichment of GAPDH over the control IgG signal.
